# Validating an advanced smartphone application for thermal advising in cold environments

**DOI:** 10.1007/s00484-023-02553-w

**Published:** 2023-10-14

**Authors:** Jakob Eggeling, Christofer Rydenfält, Amitava Halder, Jørn Toftum, Lars Nybo, Boris Kingma, Chuansi Gao

**Affiliations:** 1 Thermal Environment Laboratory, Division of Ergonomics and Aerosol Technology, Department of Design Sciences, Lund University, Sölvegatan 26, 223 62 Lund, Sweden; 2 Department of Experimental Medical Science, Integrative Physiology, Lund University, Sölvegatan 19, 22184 Lund, Sweden; 3 Department of Environmental and Resource Engineering, Technical University of Denmark, 2800 Kongens Lyngby, Denmark; 4 Department of Nutrition, Exercise, and Sports, University of Copenhagen, Nørregade 10, 1165 Copenhagen, Denmark; 5 TNO, The Netherlands Organization for Applied Scientific Research, Unit Defence, Safety & Security, Kampweg 55, 3769 DE Soesterberg, the Netherlands

**Keywords:** Cold stress, Prediction model, Safety and health, Cold environment

## Abstract

The ClimApp smartphone application was developed to merge meteorological forecast data with personal information for individualized and improved thermal warning during heat and cold stress and for indoor comfort in buildings. For cold environments, ClimApp predicts the personal thermal stress and strain by the use of the Insulation REQuired model that combines weather and personal physiological data with additional consideration of the Wind Chill index based on the local weather forecast. In this study, we validated the individualized ClimApp index relative to measurements and compared it with the Universal Temperature Climate Index (UTCI). To this aim, 55 participants (27 females) were exposed to at least 1 h in an outdoor environment of 10 °C or below (average 1.4 °C air temperature, 74.9% relative humidity, and 4.7 m/s air velocity) inputting their activity level and clothing insulation as instructed by ClimApp. The UTCI and ClimApp indices were calculated and compared to the participants’ perceived thermal sensation. The ClimApp index root mean square deviation (RMSD) was below the standard deviation of the perceived thermal sensation which indicates a valid prediction and the UTCI RMSD was higher than the standard deviation which indicates an invalid prediction. The correlation of ClimApp and UTCI to the perceived thermal sensation was statistically significant for both models.

## Introduction

As highlighted in the latest Intergovernmental Panel on Climate Change (IPCC) Assessment Report 6, climate change implies that the average global temperatures increase, but also that extreme rainfalls and cold may increase and potentially expose humans to harmful thermal stress (Masson-Delmotte et al. 2021; Pörtner et al. 2022). Countries that already experience severe heat stress during peak months are expected to have their production rates decreasing even further (Dunne et al. 2013). Mora et al. (Mora et al. 2017) identified that almost a third of the global population is exposed to deadly climatic conditions for more than 20 days per year. According to their simulations, the eastern Pacific region is the most exposed to this extreme heat (Mora et al. 2017). Even though global temperatures increase along with the apparent risks of future heat stress, cold weather will continue to be a dominant thermal contributor to mortality and morbidity in temperate zones (Hajat et al. 2014; Holmér et al. 2012; Ebi and Mills 2013; Analitis et al. 2008). By increasing the resilience of societies and companies, the impact of climate change may be reduced. Factors that can improve resilience from a thermal standpoint may be suitable clothing, availability of hydration, shading or shielding, physical fitness, acclimatization, education, advisory tools, or warning systems (Petersson et al. 2019; Casanueva et al. 2019; Morabito et al. 2019; Lemke and Kjellstrom 2012).

**Fig. 1:**
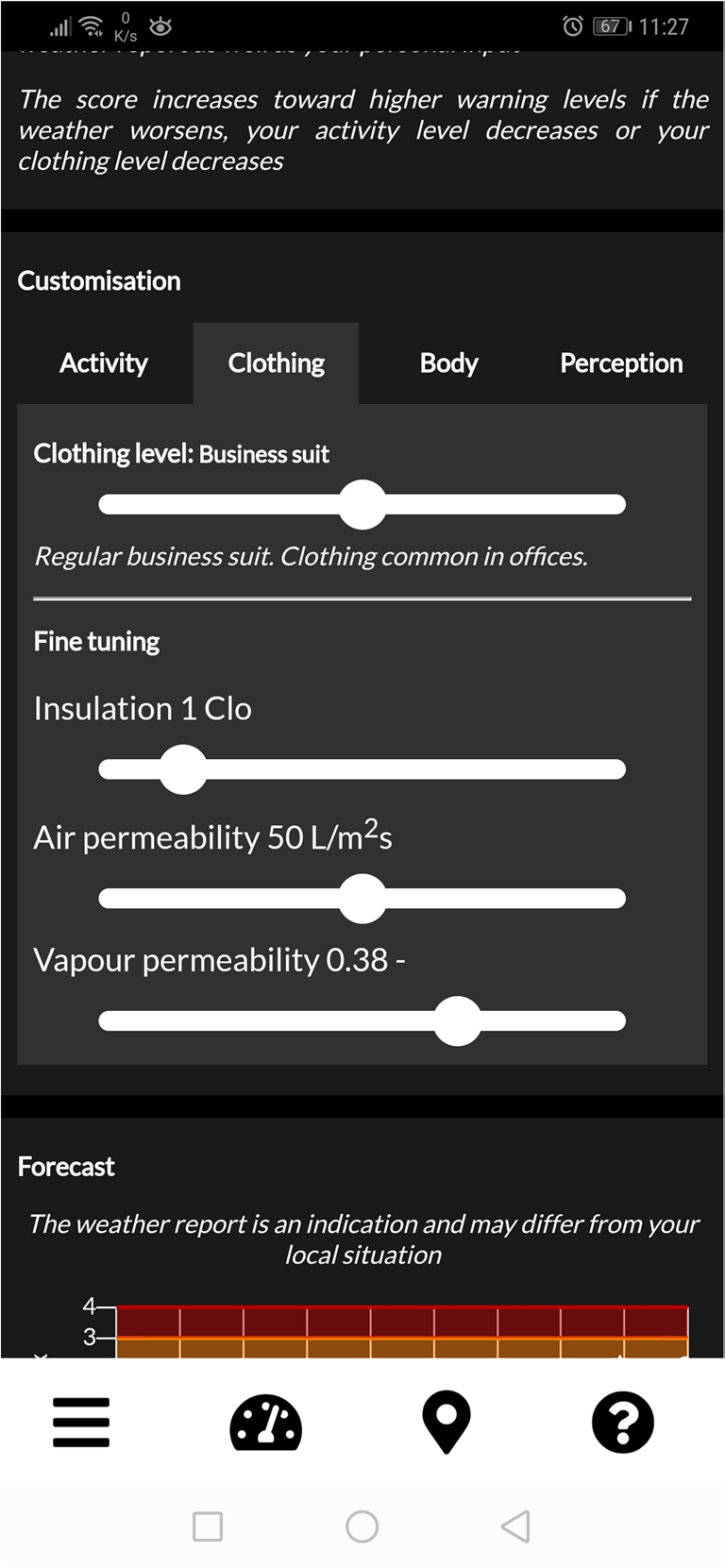
The ClimApp clothing level selection is done using five preset levels in the Customisation panel in the app. Similarly, the activity is selected using five preset levels

The application being evaluated in this study, ClimApp, is a smart phone application (app) that predicts individual thermal stress and strain by also considering activity level and clothing insulation. By including personal factors, ClimApp can assist on an individual level as thermal stress affects individuals differently depending on the microclimate. ClimApp predictions are designed to cover the temperature interval −50 °C to +50 °C which are the valid temperature ranges of the underlying models, including the comfort range of temperatures usually encountered in indoor environments. ClimApp uses a combination of several human thermal models: Predicted Heat strain (PHS) and WBGT in hot environments, Predicted Mean Vote (PMV) in moderate thermal indoor environments, and Insulation REQuired (IREQ) and Wind Chill Temperature (WCT) in cold environments (Kingma et al. 2021). This combination of models to predict the thermal stress and strain derives into a single unit and presents the user with an Index called the ClimApp index ranging from +4 (very high heat risk) to −4 (very high cold risk) where 0 (little or no risk of thermal stress) is an ideal environment. The weather data is gathered from open-source weather forecasts. The user also needs to provide the app with information on their planned activity and clothing, body weight and height, and acclimatization to heat (Kingma et al. 2021), which together with the weather data forms the basis for the thermal stress prediction. In the app, the user clothing and activity is set to a level 1-5 which corresponds to the user intended clothing ensemble and activity. The clothing levels range from “Summer clothing” to “Winter clothing” and the activity levels range from “Rest” to “Intense” with explanatory text to each level, the clothing levels are implemented from ISO 7243 (ISO_7243 2017) and the activity levels with the determined metabolic rate are implemented from ISO 8996 (ISO_8996 2021). The insulation value for each clothing level is displayed in the app and the user is able to fine-tune to clothing properties, if necessary, which can be seen in Fig. 1. Based on the prediction, the user receives advice and recommendations on how they can reduce the risk of thermal stress. Such advice can be hydrating properly, taking frequent rest, performing heavy or laborious tasks during colder periods of the day, or dressing in less or additional layers (Kingma et al. 2021; Eggeling et al. 2022).

When assessing thermal stress, each situation is different, which calls for situational predictions. For a wider and more general warning system, it can be suitable to provide a warning based on the prevailing and upcoming weather at the user’s geographical location (Morabito et al. 2011). Indices such as the Universal Temperature Climate Index (UTCI) combines the available weather parameters into one index value that is straightforward to apply and represents the climate very well compared to more simple indices (Blazejczyk et al. 2012). The strength of UTCI compared to indices derived solemnly from weather variables is that it also includes a fixed activity and an adaptive clothing value based on the weather variables such as wind (Błażejczyk et al. 2013; Havenith et al. 2012). UTCI is getting more commonly used in thermal stress studies as it quantifies thermal stress based on the existing weather parameters, but it does not allow for personalized thermal stress prediction as the activity and clothing in the model cannot be changed. There can be benefits of including in a prediction more detailed personal information, such as planned clothing and activity that vary from individual to individual, and when work conditions are governed by legislation or cultural norms (Lundgren et al. 2014). In many workplaces, specific work clothes including protective layers must be worn regardless of prevailing weather, which requires further attention when assessing thermal stress (Kuklane et al. 2015). Protective clothing is not covered in UTCI (Gao et al. 2018) but is considered in ClimApp with the integrated WBGT (Sakoi et al. 2018; ISO_7243 2017). The need of a decision-support system in cold environments has been concluded in recent research (Austad et al. 2018; Petersson et al. 2019; Xu et al. 2021).

The usability of ClimApp was evaluated to investigate how the users interacted with the tool and what challenges existed. The usability was tested in a usability lab and in the field where first-time users completed tasks in ClimApp related to navigating in the app, perceived ease of use, and perceived usefulness (Eggeling et al. 2022). The usability testing proved fruitful in that the core concept of evaluating individual thermal stress was clearly understood and proved no issues for the user, but more complicated tasks such as changing indoor parameters or assessing remote locations were problematic (Eggeling et al. 2022).

The aim of this study was to assess the thermal stress prediction of ClimApp in cold environments based on thermal perception. ClimApp was previously validated in warm to hot environments ranging between 14.5-34.8 °C but not in the cold (Folkerts et al. 2021). The perception and prediction were also compared to UTCI to evaluate the sensitivity of the prediction to the personalization factor.

## Methods

### Participants

The power calculation pointing out the number of required participants was computed with the GPower software to ensure sufficient power to detect statistical significance (Faul et al. 2007). In order to test the model prediction of ClimApp, the “*F* test linear multiple regression model, R2 deviation from zero” was used to calculate the sample size. Existing studies evaluating UTCI reported using a medium effect size (Nie et al. 2022). With a chosen medium effect size of *f*^2^ = 0.15 (Cohen 2013), *α* = 0.05, power = 0.80, and the number of predictors being 1, the total sample size was computed to be 55.

Fifty-five participants were therefore recruited and briefed about the study aim and methodology over the telephone or through e-mail correspondence. The mean age (standard deviation), weight, and height of the participants were 32.0 (11.2) years, 73.6 (16.4) kg, and 175.2 (10.1) cm. The selection criteria were that the participant was 18 years or older and frequently spent at least 60 consecutive minutes outdoors for outdoor activities during the winter months (air temperature lower than 10 °C). Participants were instructed to abstain from participating if they had issues with being outdoor in the cold or had medical conditions, which could alter their perception of thermal sensation. The test was conducted remotely due to COVID-19 restrictions. The participants were given a written instruction with information regarding the study outcome, how the study is conducted, and contact information to the researchers. Additionally, if the participants experienced any difficulties, they were encouraged to contact the researchers.

The experimental procedure required the participants to spend at least 60 min in an outdoor environment of their own choice. The time requirement was set to at least 60 min to allow sufficient exposure and physiological responses to the cold environment. The participants were instructed to avoid heating sources such as bonfires or heat exhausts. The participants dressed up in their clothing, planned their physical activity, and entered these values into ClimApp to get a prediction. Each participant then recorded what ClimApp predicted of their thermal stress to be in 60 min and then started the outdoor exposure. The participants also recorded the UTCI Equivalent Temperature prediction as well as the weather conditions during the test. After the 60 min had passed, the participants recorded their own perceived thermal sensation and submitted all recordings through a web-based survey after the test. The participant clothing insulation and activity input data are retrieved from the surveys and the UTCI clothing is calculated according to Havenith et al. (2012) based on the UTCI Equivalent Temperature retrieved from the survey and the UTCI activity is a fixed value in the model.

### Ethical considerations

Each participant signed a consent form and informed us that they had read and understood the terms of the study. The participants had the rights to withdraw from the study at any time without stating any reason and they had the opportunity to ask for the data to be deleted without questioning. The participants were informed not to make any decisions based on ClimApp that were contradictory to their own choice.

### Data analysis and processing

**Fig. 2:**
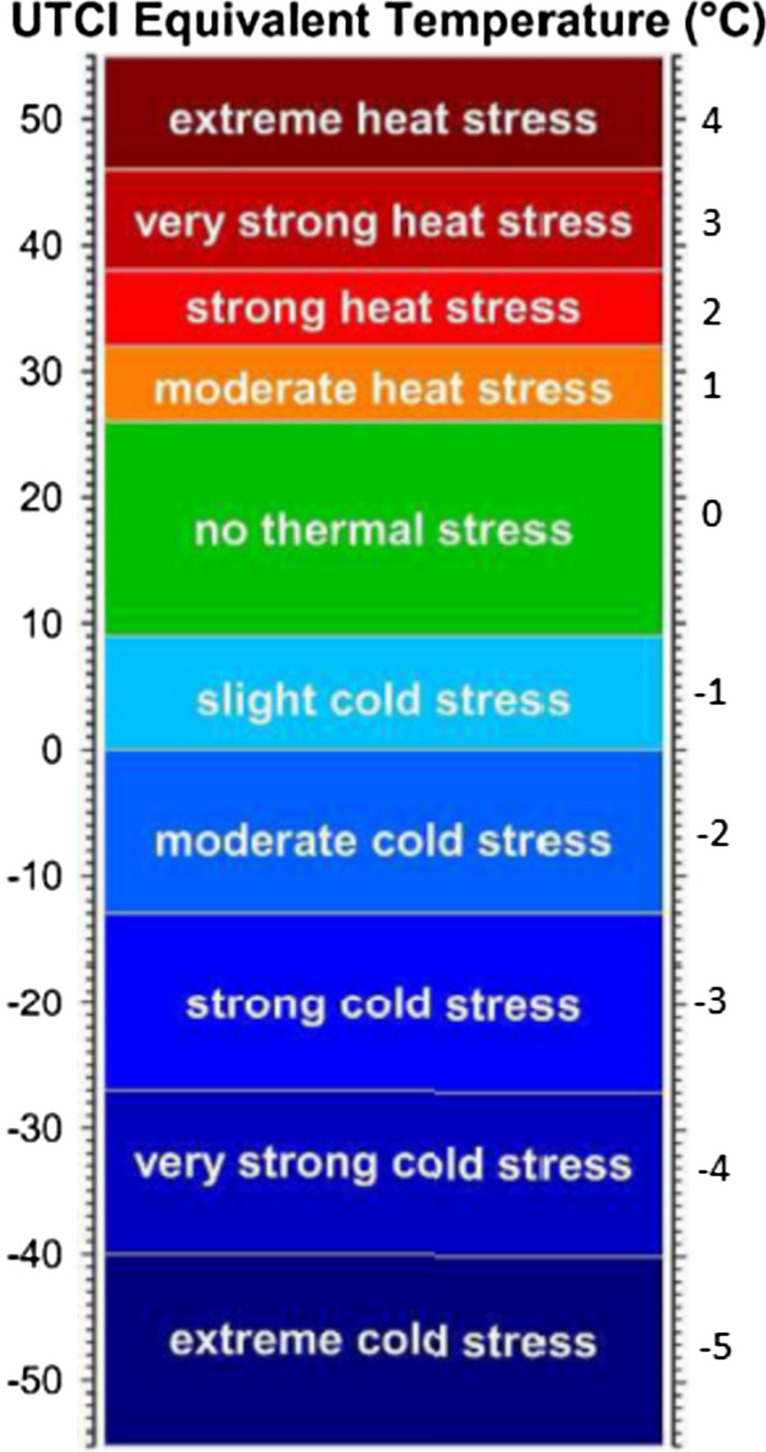
The UTCI scale with corresponding index values. The image is modified from Błażejczyk et al. (2013) and Broede et al. (2013) and the index levels are inferred as those of similar indices such as PMV (ISO_7730. 2005)

The processing of data and statistical analyses were performed in Excel 2016 (Microsoft Corporation, U.S.A.) and RStudio version 1.4.1717 (RStudio, USA.). The root mean square deviation (RMSD) calculation was performed to assess the validity of the ClimApp index compared to the user perception (Haslam and Parsons 1994; Bogerd et al. 2010). This was also done for UTCI, which was indexed into the thermal stress levels given by Błażejczyk (Błażejczyk et al. 2013; Broede et al. 2013). This indexed scale uses similar thermal sensation levels as the ClimApp index (Kingma et al. 2021), see Fig. 2, with four heat stress levels and five cold stress levels while ClimApp uses four levels for both heat and cold stress. The stress levels are related to different physiological responses which are listed in the introduction to UTCI by Błażejczyk et al. (Błażejczyk et al. 2013). During data analysis, the reported UTCI Equivalent Temperature result was matched to the corresponding thermal stress level, where a UTCI Equivalent Temperature of −10 corresponds to the −2 level (Fig. 1). In the analysis, the indexed UTCI was used primarily for evaluation and the original equivalent temperature was used to confirm that the indexed results were reliable. A RSMD value lower than the standard deviation of the perceived score is considered a good fit for the prediction (Xu et al. 2005; Castellani et al. 2007).

## Results

The participants completed the test in the specified test conditions for the duration of 60 min. Table 1 shows aggregated values characterizing the exposure during this period. The average environmental condition was a temperature of 1.4 °C, a relative humidity of 74.9%, and air velocity of 4.7 m/s.

**Table 1:** Mean, standard deviation, and range of the test conditions

	Temperature (°C)	Relative humidity (%)	Air velocity (m/s)	Wind Chill Temperature (TWC)
Mean	1.4	74.9	4.7	−2.2
SD	5.6	16.1	2.6	6.5
Range	−13.0–10.0	46.0–100.0	0.0–12.0	−19.1–10.0

Table 2 shows the clothing insulation and metabolic rates recorded by the participants. The most common activity was walking (moderate activity) and participants mostly wore double-layered clothing. The UTCI activity value was fixed to 135 W/m^2^ and the clothing insulation was determined based on temperature (Havenith et al. 2012). The average ClimApp input values for both activity and clothing, as entered by the participants, were higher than the UTCI estimated values (Table 2).

**Table 2:** Mean, standard deviation, and range of activity and clothing used for calculation of the ClimApp and UTCI indices

	ClimApp activity (W/m^2^)	UTCI activity (W/m^2^)	ClimApp clothing (clo)	UTCI clothing (clo)
Mean	148.2	135	1.6	1.3
SD	30.6	0	0.5	0.1
Range	57–260	135–135	0.5–2.5	1.1–1.5

The validity of ClimApp was tested by RMSD of the predicted ClimApp index, which was also used to test the UTCI (Table 3). The RMSD of the ClimApp index (1.05) was found smaller than the standard deviation of the perceived score (1.31) of thermal sensation and thus fell in the criteria for a valid prediction. The obtained RMSD of UTCI was 15.6, which was higher than the SD of the perceived score of thermal sensation and it is not within the criteria for a good prediction.

**Table 3:** RMSD for the ClimApp index and UTCI compared to the standard deviation (SD) of the perceived score of the participants. The mean value and the range are presented for the indices and the perception

	Perception	ClimApp	UTCI
RMSD		1.05	15.65
Mean	0.27	0.39	−1.48
SD	1.31	0.84	0.93
Range	−3–2	−3–2	−3–0

**Fig. 3:**
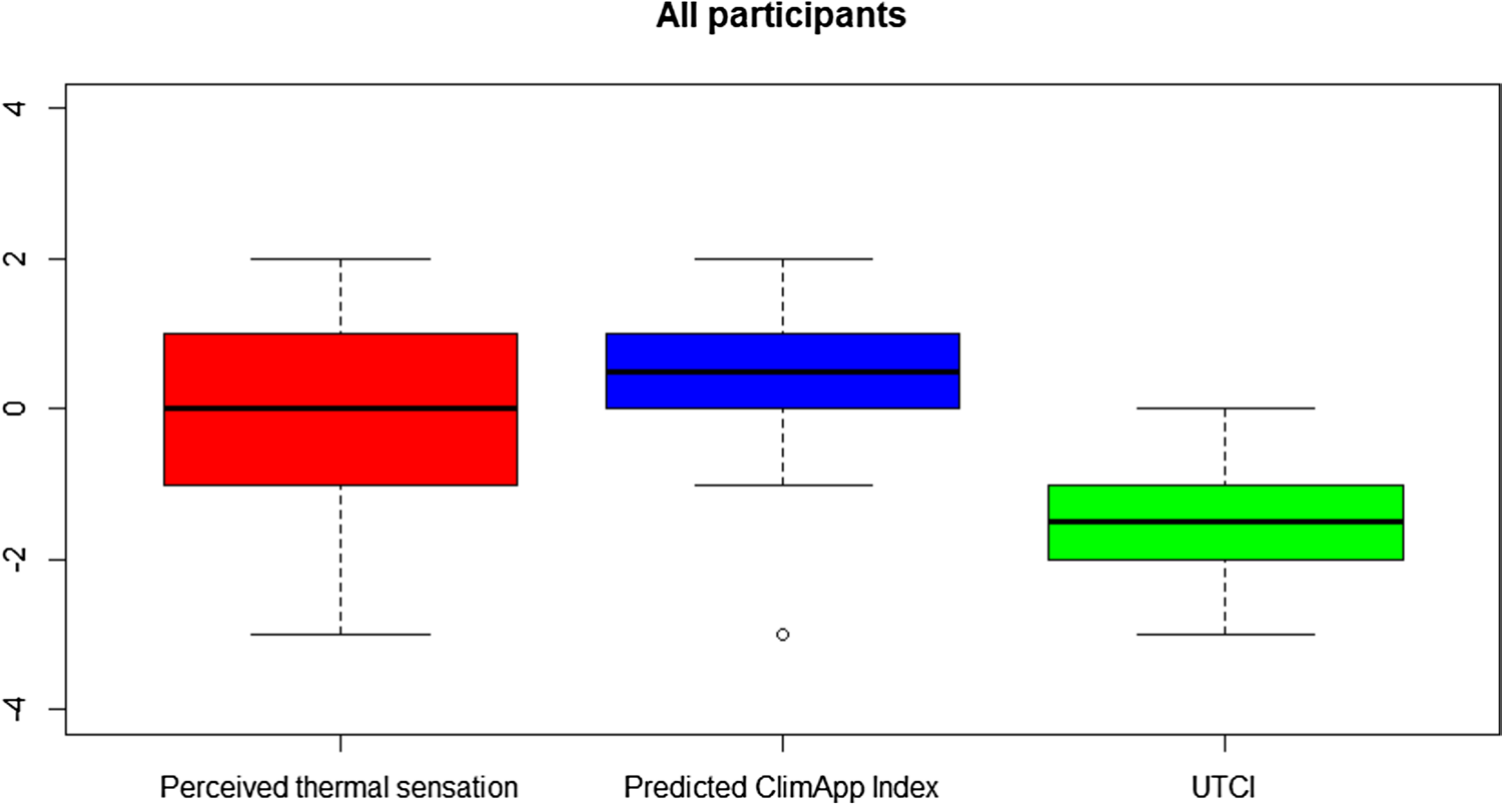
Boxplots of the perceived thermal sensation, the predicted ClimApp index, and the UTCI. The boxes span from the first to the third percentile with a solid black line representing the median value. The whiskers represent the range while the dot in the boxplot for the predicted ClimApp index has a single outlier

The correlation was tested between the perceived score and all available variables using a Pearson’s correlation test where significance levels are noted as: *p < 0.05, **p < 0.01, and ***p < 0.001. Both the ClimApp index (**) and the UTCI (**) significantly correlated with the perceived score, as did temperature (*) and wind chill temperature (*). The results of the perceived thermal sensation, the ClimApp index, and the UTCI are visualized as boxplots in Fig. 3.

**Fig. 4:**
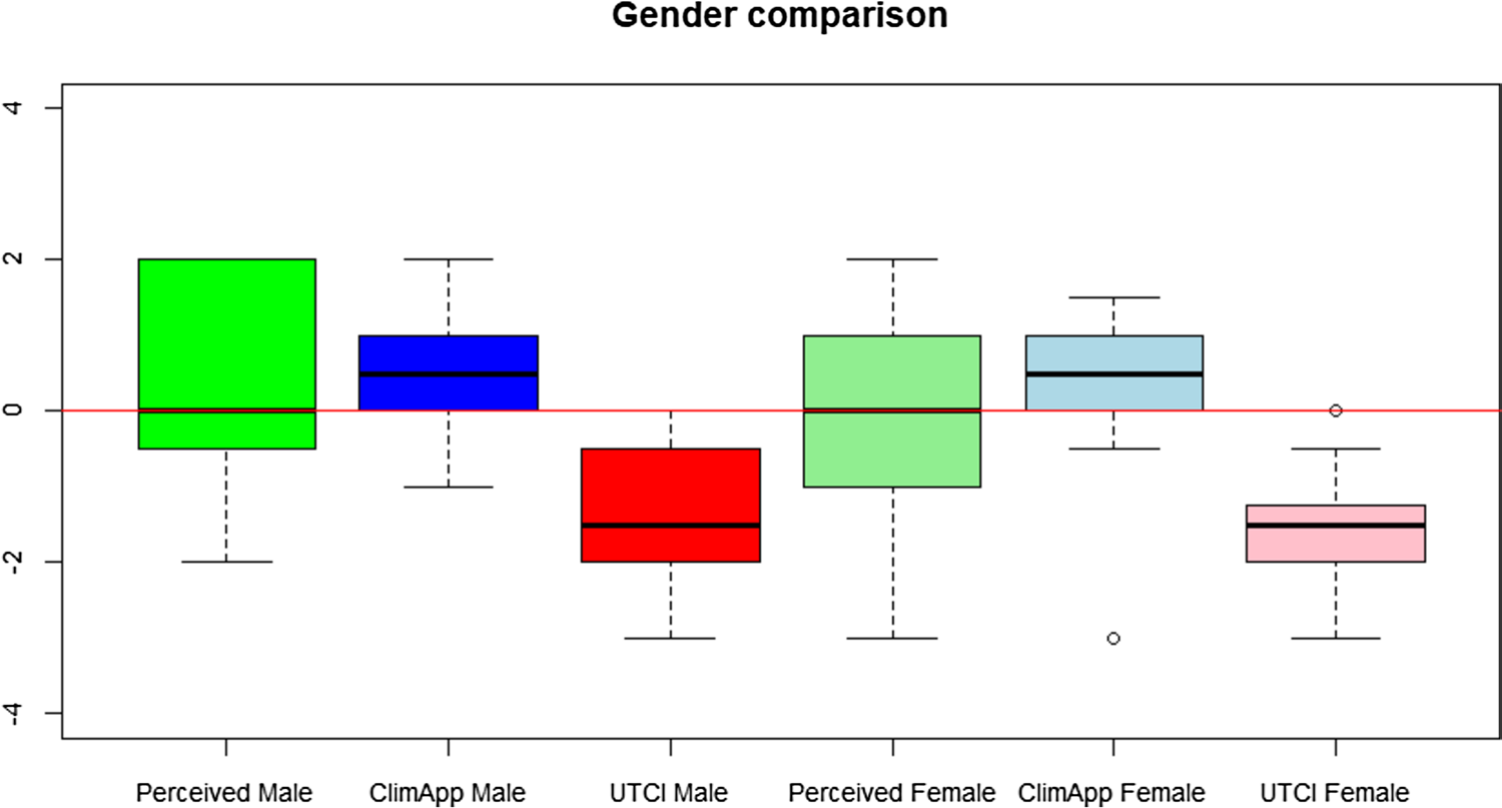
The different values are represented by boxplots for both male and female with the same representation structure of boxplots as in Fig. 3. A thin red line highlights a thermoneutral value. For clarification of the labels, perceived male corresponds to the perceived thermal sensation of the male group and ClimApp male corresponds to the ClimApp prediction index of the male group

Figure 4 shows the perceived thermal sensation, the predicted ClimApp index and the UTCI of both male and female participants. None of the indices differed between the genders. The average predicted ClimApp index for females was 0.41 and 0.38 for males while the average perceived score was 0.07 for females and 0.46 for males. No differences were found in activity or clothing between the genders. The average UTCI Equivalent Temperature for all participants was −2.13 °C which corresponds to −2 on the scale in Fig. 2. The average UTCI Equivalent Temperature of −2.13 °C is lower than the average indexed UTCI value of −1.52.

## Discussion

The findings of this study show that thermal stress, predicted from integration of weather forecasts and human heat balance models, can assist individuals when they prepare for outdoor exposure in cold environments. The ClimApp prediction only slightly overestimated the thermal stress as most of the predictions were consistent with the participants’ perceived thermal sensation. On the contrary, the UTCI over-predicted the thermal stress. This may be because of the fixed value of activity and the estimated temperature dependent clothing. These were both below the values entered by the participants, which implies the benefit of letting individuals enter values for activity and clothing.

Both the ClimApp index and the UTCI significantly correlated with the perceived thermal sensation of the users, which indicates that both provide meaningful results. The ClimApp index also scored a RMSD below the SD of the perceived thermal sensation and the mean ClimApp index was very close to the perceived thermal sensation. This suggests that the index is well suited for predicting thermal sensation in cold environments. UTCI on the other hand scored a RMSD value far above the SD of the thermal sensation and the mean value was much lower than the thermal sensation reported by the participants. The correlation was significant for UTCI indicating that the model captured the trend. Adding conditions in UTCI that increase insulation and activity in colder environments could be favorable according to the results found in this study.

The most common activity chosen in ClimApp was moderate (walking), corresponding with a metabolic rate of 150 W/m^2^. The mean of the whole group of participants was 148 W/m^2^_,_ considerably higher than the metabolic rate of 135 W/m^2^ used to calculate UTCI. The clothing insulation used in UTCI is derived from the weather input and for these test conditions resulted in 1.3 clo. Most participants chose the double-layered clothing in ClimApp, which corresponds with 1.5 clo. However, the group average was 1.6 clo, as several participants chose the winter clothing level (2.5 clo). When assessing the cases of poor prediction by ClimApp, there was no indication of wrong selection of clothing or activity level. A few participants selected extreme activities, such as resting or intense, and ClimApp predicted the thermal stress for these participants just as well as for the moderate activity level.

The results indicate a need to evaluate modifications of the UTCI clothing algorithm in cold environments, as the low activity and low clothing insulation lead to a disconnect between the index and actual outdoor exposure. If the value for activity in UTCI was set to 150 W/m^2^ similar to the average ClimApp input, and the UTCI clothing algorithm was altered to increase the clothing insulation in these environments, UTCI would probably better predict the exposure in this condition. The conversion of the UTCI into a scale like that of ClimApp should be treated with extra attention as the UTCI scale is asymmetrical and each level has different interval ranges. However, when assessing the average UTCI Equivalent Temperature of −2.13 °C, it is still in the moderate cold stress level (−2). This moderate cold stress is lower than the indexed UTCI value of −1.52 reported in this study.

There was a slight difference in perceived thermal sensation between male and female participants. The difference was not statistically significant, but it is an indication that gender differences play a part in thermal sensation. No differences were found in activity or clothing between the genders that could address the difference in perceived thermal sensation. ClimApp in its current state does not account for gender differences but future development of the app could investigate and incorporate such differences.

It is important that a thermal stress warning system provides its audience with relevant advice and warnings (Flouris et al. 2018). Ethical decisions and occupational health and safety regulations require accurate information to provide appropriate limits. Based on these limits, work leaders can decide if the production targets are feasible or if there are elevated health risks that need to be lowered. Abandoning production targets have direct and often known financial costs, while evaluation of occupational health requires further evaluation. It is, therefore, beneficial to provide decision makers with easily accessible exposure assessment and advice on mitigation strategies of thermal stress.

The study was conducted during the pandemic lockdown. The participants had to be enlisted, briefed, and complete the study remotely to avoid putting both the researchers and participants at risk. However, this limitation was partly mitigated by continuous contact with each participant to make sure that the purpose and procedure were clear to the participant. The current validation was based on participant perception of their thermal sensation. However, the thermo-physiological responses, such as skin and core temperatures, were not measured and are considered to be limiting factors. Further evaluation of the ClimApp index should address such limitations and can be tested in a smaller sample study.

## Conclusions

This study shows that the individualized thermal stress ClimApp index well predicted the perceived thermal sensation of the study participants. The personalization aspect of changing clothing and activity by the user is shown to improve the prediction accuracy of ClimApp compared to that of the non-personalized UTCI. Both the ClimApp index and UTCI correlated significantly with the observed thermal sensation, but only the ClimApp index had a RMSD value below the standard deviation which indicates a valid model. A tool such as ClimApp that provides both heat and cold stress warnings may be valuable as it is freely available and easily accessible for a large part of the global population of smart phone users when facing more frequent extreme weather events due to climate change.
